# The influence of socioeconomic position on patient-reported outcome measures following hip fractures – a register-based observational study on 35,206 patients from the Norwegian hip fracture register 2014–2018

**DOI:** 10.1186/s12955-025-02377-9

**Published:** 2025-05-04

**Authors:** Cato Kjaervik, Jan-Erik Gjertsen, Eva Stensland, Jan Abel Olsen, Christer Kjaervik, Odd Soereide

**Affiliations:** 1 Department of Surgery, Nordland Hospital Trust, Vesteraalen Hospital, Ivar Bergsmos gt 3, Stokmarknes, 8450 Norway; 2 Department of Clincal Medicine, UiT, The Arctic University of Norway, Tromsø, 9012 Norway; 3 Department of Orthopaedic Surgery, Norwegian Hip Fracture Register, Haukeland University Hospital, Bergen, 5009 Norway; 4 Department of Clinical Medicine, University of Bergen, Bergen, 5007 Norway; 5 Department of Community Medicine, UiT, The Arctic University of Norway, Tromsø, 9012 Norway; 6 Centre for Clinical Documentation and Evaluation (SKDE), Northern Norway Regional Health Authority, Tromsø, 9012 Norway

## Abstract

**Background:**

Hip fractures are a significant public health concern due to increasing numbers, high mortality and negative impact on health-related quality of life (HRQoL). Socioeconomic position (SEP) affects various health outcomes, but the specific impact on HRQoL and satisfaction after hip fracture remains underexplored. This study assesses whether education and household income influence patient-reported outcome measures (PROMs) after hip fractures, measured by three visual analog scales: EQ-VAS, pain-VAS, and satisfaction-VAS.

**Methods:**

This was a nationwide retrospective cohort study using linked data from the Norwegian Hip Fracture Register and Statistics Norway. PROMs assessed at 4, 12, and 36 months postoperatively in 35,206 hip fracture patients from 2015 to 2018 were included. The SEP data included household income and education levels. Covariance analyses were conducted to evaluate differences in mean VAS scores for general health (EQ-VAS), pain from the operated hip (Pain-VAS), and satisfaction with the result of the operation (Satisfaction-VAS). Analyses adjusted for age, sex, vital status, cognitive impairment, treatment type, and education or income when not used as independent variable.

**Results:**

The study included 23,649 women (67.2%) and 11,557 men (32.8%) with median age 83 years. Lower education was linked to worse EQ-VAS and Pain-VAS scores at all follow-ups and to lower Satisfaction-VAS at 12 and 36 months in both unadjusted and adjusted analyses. Lowest level of income had significant lower EQ-VAS at all follow-ups, lower Pain-VAS at 12 months, and lower Satisfaction-VAS at 4 months. There were increasing differences in mean VAS-scores during follow-up. At 36 months the adjusted differences in mean EQ-VAS between highest and lowest level of income was − 2,51 (-4.04 -0.99). Differences across education levels were even stronger associated; -3.58 (-5.19 to -1.98). Mean differences in Pain-VAS between medium and low education compared to high were 4.30 (2.91 to 5.69) and 5.58 (4.08 to 7.08), respectively. Lower levels of education also had significant negative differences in Satisfaction-VAS at 36 months follow-up -4.06(-5.86 to -2.26).

**Conclusions:**

Lower education and income were significantly associated with worse HRQoL and satisfaction after hip fracture. The clinical relevance of these findings warrants further investigation. Addressing SEP disparities should be integral to hip-fracture care strategies aiming to improve postoperative outcomes.

## Background

Hip fractures constitute a major public health concern because of their high incidence and significant negative effects on patients’ health [1, 2]. Almost 9,000 individuals in Norway suffer from hip fracture annually, and this number is expected to increase in the future due to a growing number of elderly patients at risk, despite ongoing efforts to prevent hip fractures in this age group [3–5]. Health-related quality of life (HRQoL) is often significantly compromised following a hip fracture [6, 7], leading to decreased self-perceived health, increased pain, and disability [8].

Socioeconomic position (SEP) is a well-established determinant for various health outcomes, with lower SEP being linked to more complex health problems and negative consequences for HRQoL [9]. This relationship is partly mediated by health behaviors, as higher SEP is strongly linked to healthier lifestyles [10, 11]. However, the specific associations between SEP and health outcomes in hip fracture patients remain underexplored. Investigating these associations is crucial, as understanding the role of SEP could inform targeted interventions to improve recovery and quality of life for hip fracture patients across different socioeconomic backgrounds, and aid prevention of new fractures.

This study will shed light on the interplay between SEP and PROMs after surgically treated hip fractures and the implications for healthcare provision, including the broader issue of health inequalities. The study is grounded in the “A Conceptual Framework for Action on the Social Determinants of Health” [12], which postulate that SEP is a key determinant of health outcomes. SEP influences health through multiple pathways, including access to healthcare, health behaviours, and environmental exposures. The study also draws on the “Operational framework for monitoring social determinants of health equity” [13], which states that effective monitoring of social determinants of health (SDH) is critical to understanding and addressing health inequities. The aim of the study was to assess whether socioeconomic factors, specifically education and household income, have an impact on PROMs (measured by self-perceived health and pain) and satisfaction of the surgical result.

## Methods

Using linked data from the Norwegian Hip Fracture Register (NHFR) and Statistics Norway (SN), we performed a nationwide (5.3 million inhabitants at the end of the study period in 2018) retrospective cohort study of prospectively collected data.

### The Norwegian hip fracture register

Data on hip fracture patients (ICD-10 [14] codes S72.0-S72.2) operated on in Norwegian hospitals have been collected by the NHFR since 2005 [15], and baseline patient characteristics (sex, age, American Society of Anesthesiologists physical status (ASA) class [16], type of surgery given and presence of chronic cognitive impairment) were extracted. Hip fractures treated with total hip arthroplasty were recorded in the Norwegian Arthroplasty Register and subsequently imported to the NHFR. Type of treatment was categorized into Osteosynthesis (i.e. cannulated screws, dynamic hip screws, Intramedullary nails), Hemiartrhoplasty, Total hip arthroplasty and Other (combined interventions or rare treatment methods).

The date of death was imported to the NHFR from the National Population Register. Patient Reported Outcome Measure (PROM) questionnaires were sent from the NHFR to all living patients at 4, 12, and 36 months postoperatively. No reminders were sent to non-responders. Patients treated with THA (*n* = 1,694) only completed the four-month PROM questionnaire.

The PROM questionnaire included three measures of health, all of which used a visual analog scale (VAS). First, the EQ-VAS is based on respondents’ direct valuations of their overall health-related quality of life (HRQoL) on a scale that ranges from 0 (*worst imaginable health*) to 100 (*best imaginable health*) [17]. Second, the Pain-VAS measuring the patients´ average self-reported pain from the operated hip the last month before the defined follow-ups, ranging from 0 to 100 (100 represents the worst possible pain). Third, the Satisfaction-VAS measuring satisfaction with the result after the surgical treatment of the fractured hip and is reported on a scale from 0 to 100, where 0 represents the most satisfactory score. For analytical purposes and presentation in the paper, the scale was inverted, i.e., 100 represents most satisfied, and 0 represents least satisfied. Note that the Satisfaction-VAS explicitly refers to ‘the result after the surgical treatment’, i.e. its outcome. We therefore categorized it as a PROM-variable, rather than a PREM that refers to *experience* with the treatment per se.

The completeness of reporting in the NHFR has been regularly evaluated and was 88% for osteosynthesis, 95% for hemiarthroplasties, and 88% for total hip arthroplasty in 2015-2016 [18]. This completeness evaluation was performed in the middle of the inclusion period, and we assume that these figures are valid for the data used in this study.

### Statistics Norway

We acquired individual socioeconomic position data from Statistics Norway (SN), including household income and the highest level of education attained. Household income, defined as the household’s total taxable income (including wages, social benefits, pensions, etc.) in the year prior to the injury, was divided into three equally sized groups: low (< 20,500 Euro), medium (20,500 − 36,600 Euro), and high (> 36,600 Euro-). Converted from Norwegian crowns (NOK) January 2025. The International Standard of Classification of Education classified educational levels into three levels [19]: low, which represents lower secondary education; medium, which represents upper secondary to short-cycle postsecondary education; and high, which represents bachelor’s degree and higher education.

**Fig. 1:**
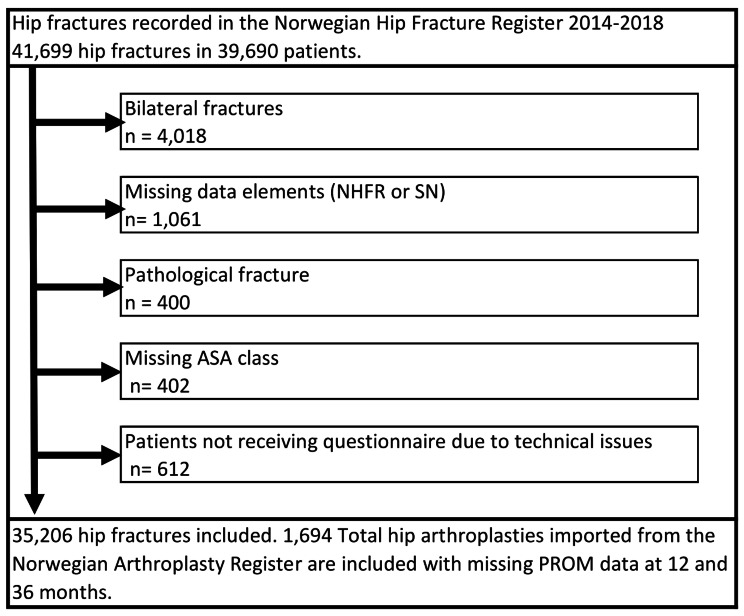
Patient inclusions and exclusions Legend: Norwegian Hip Fracture Register (NFHR) - Statistics Norway (SN) - American Society of Anesthesiologists physical status class (ASA)

As of 31 December 2019, the NHFR included data on 41,699 fractures with a minimum of one year of follow-up from 1 January 2014 to 31 December 2018. Since it would have been challenging to maintain distinct follow-up periods for fractures occurring on both sides, patients with bilateral fractures were excluded during the inclusion period (*n* = 4,018 in 2,009 patients). These patients were of slightly higher age and exhibited greater comorbidity, as measured by ASA classification and the presence of cognitive impairment, compared to the study population. Furthermore, patients with pathological fractures (*n* = 400), patients with missing data on ASA class (*n* = 402), and patients with missing data in the coupled datasets (NHFR and SN) (*n* = 1,673) were excluded (Fig. 1). Thus, data from 35,206 patients were available for analysis.

### Statistical analysis

Demographic variables are presented as absolute numbers and percentages. The time trend data are crude mean values stratified separately for the 3 categories of education and of household income. The level of significance was set at *p* < 0.05 in all analyses.

**Fig. 2:**
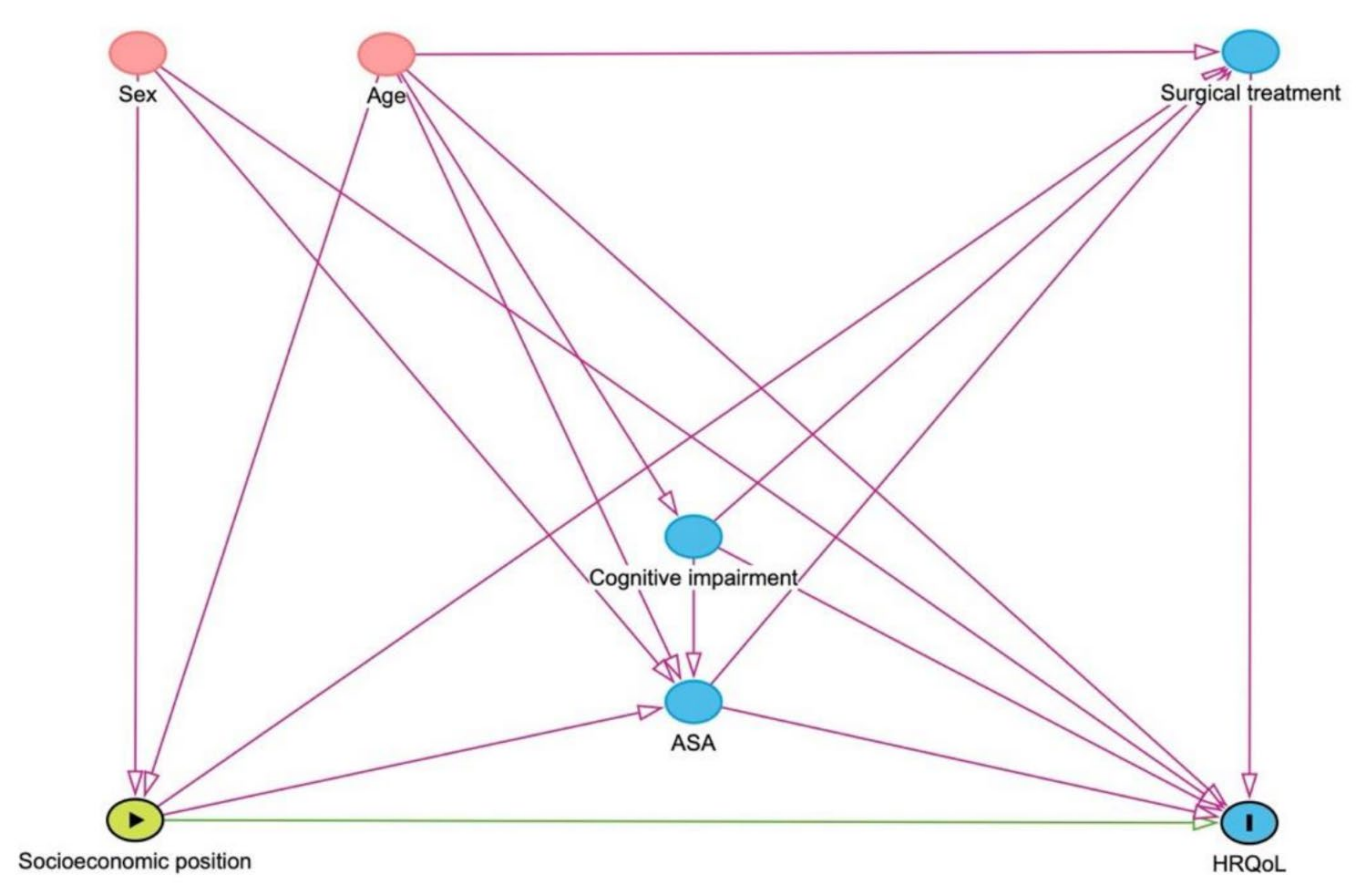
Directed acyclic graph (DAG) visualizing possible causal relationships among available covariates Legend: All included covariates presented. Socioeconomic position is represented by level of education and household income. ASA class - American Society of Anesthesiologists physical status represents observed comorbidity at surgery

To provide a simple visual representation of the potential causal relationships among the variables included in this study and to select variables to include in the statistical analyses for adjustment, we applied a directed acyclic graph (DAG) model (http://www.daggity.net) (see Fig. 2). The DAG representation is useful for determining whether a given pair of variables are independently associated and the empirical directions of effects. The DAG representation makes explicit what assumptions are being made if causal relationships are not ascertained and is particularly useful when estimating causal relationships from non-randomized studies, which are subject to confounding factors.

To assess the associations between SEP (level of education and level of household income) and the mean VAS score on the three VAS scales (EQ-VAS, Pain-VAS, and Satisfaction-VAS), we performed an analysis of covariance (ANCOVA) utilizing a generalized linear model (GLM) with the PROC GLM function in SAS Statistics. We performed six separate analyses at all three follow-ups: categories of education and household income versus EQ-VAS, Pain-VAS, and Satisfaction-VAS scores. To assess the magnitude of difference in mean VAS scores between categories of education and household income on the EQ-VAS, Pain-VAS, and Satisfaction-VAS scores at 4, 12, and 36 months, the same analysis of covariance (ANCOVA) as described before was performed. The magnitudes and confidence intervals (CIs) of estimations were calculated. Cohen´s d as measure of effect size was estimated as mean difference in the separate VAS scores divided by the mean square error. All analyses were adjusted for age, sex, ASA class, type of surgery, and presence of chronic cognitive impairment. In addition, all analyses were adjusted for the SEP factor that was not utilized as independent variable. I.e. analyses on education were adjusted for income and vice versa.

The analyses were performed using SAS/STATS for Windows v. 8.3 (SAS Institute, Cary, North Carolina, USA). The STrengthening the Reporting of OBservational Studies in Epidemiology (STROBE) guidelines were followed [20].

### Ethics, funding and conflicts of interest

The Northern Norway Regional Committee for Medical and Health Research Ethics approved the project and exempted it from the duty of confidentiality (REK 2018/1955). A data integrity assessment was conducted in accordance with the EU General Data Protection Regulation (GDPR). The project was funded by the Northern Norway Regional Health Authority (HNF1482-19). The NHFR is financed by the Western Norway Regional Health Authority. There are no competing interests to declare.

## Results

A total of 35,206 patients, 23,649 women (67.2%) and 11,557 men (32.8%), with a median age of 83 years (interquartile range: 76–90), were included in the study. At the time of injury, 23.8% of the patients had cognitive impairment, and 63.1% of the patients were classified as ASA class 3 or above. Most patients (86.4%) had a low to medium level of education (Table 1).

**Table 1:** Demographics

			Level of education						Household income (missing 42)					
			Low		Medium		High		Low		Medium		High	
	Total	%	*n*	% (of total)	*n*	% (of total)	*n*	% (of total)	*n*	% (of total)	*n*	% (of total)	*n*	% (of total)
Total	35,206	100	15,065	42.8	15,342	43.6	4,799	13.6	11,721	33.3	11,722	33.3	11,721	33.3
Sex			*n*	%	*n*	%	*n*	%	*n*	%	*n*	%	*n*	%
Female	23,649	67.2	10,888	72.3	9,973	65.0	2,788	58.1	10,217	87.2	7,535	64.3	5,877	50.1
Male	11,557	32.8	4,177	27.7	5,369	35.0	2,011	41.9	1,504	12.8	4,187	35.7	5,844	49.9
Age														
< 65	3,461	9.8	1,247	8.3	1,498	9.8	716	14.9	464	4.0	999	8.5	1,984	16.9
65–74	5,828	16.6	1,958	13.0	2,845	18.5	1,025	21.4	1,291	11.0	2,033	17.3	2,496	21.3
75–79	4,637	13.2	1,906	12.7	2,082	13.6	649	13.5	1,516	12.9	1,593	13.6	1,520	13.0
80–84	6,417	18.2	2,897	19.2	2,745	17.9	775	16.1	2,365	20.2	2,217	18.9	1,826	15.6
85–89	7,680	21.8	3,548	23.6	3,222	21.0	910	19.0	2,894	24.7	2,629	22.4	2,156	18.4
> 90	7,183	20.4	3,509	23.3	2,950	19.2	724	15.1	3,191	27.2	2,251	19.2	1,739	14.8
ASA class														
1	1,312	3.7	281	1.9	578	3.8	453	9.4	206	1.8	224	1.9	876	7.5
2	11,696	33.2	4,575	30.4	5,277	34.4	1,844	38.4	3,622	30.9	3,805	32.5	4,247	36.2
3	19,420	55.2	8,868	58.9	8,329	54.3	2,223	46.3	6,885	58.7	6,696	57.1	5,826	49.7
4 + 5	2,778	7.9	1,341	8.9	1,158	7.5	279	5.8	1,008	8.6	997	8.5	772	6.6
Chronic cognitive impairment														
Yes	8,396	23.8	3,877	25.7	3,549	23.1	970	20.2	3,149	26.9	2,923	24.9	2,318	19.8
No	26,810	76.2	11,188	74.3	11,793	76.9	3,829	79.8	8,572	73.1	8,799	75.1	9,403	80.2
Type of treatment														
Osteosynthesis	19,347	55.0	8,386	55.7	8,308	54.2	2,653	55.3	6,543	55.8	6,290	53.7	6,492	55.4
Hemiarthroplasty	13,688	38.9	5,924	39.3	6,019	39.2	1,745	36.4	4,745	40.5	4,832	41.2	4,103	35.0
Total hip arthroplasty	1,694	6.7	543	2.6	801	5.2	350	7.3	275	2.4	449	3.8	958	8.2
Other	477	1.4	212	1.4	214	1.4	51	1.1	158	1.4	151	1.3	168	1.4

Men represented 32.8% of the population, but they were better educated and had higher household income than women (41.9% of the men were in the highest education group and 49.9% in the top household income group). The highest educated patients had less comorbidity than the lowest educated patients (5.8% vs. 8.9% with ASA 4 + 5 respectively). Similarly, patients in the highest household income group had less comorbidity than patients in the lowest household income group (66.8% vs. 15.7% had ASA class 1 resepectively). In total 6.7% were operated with a total hip arthroplasty. Patients in the high education group and the high household income group were more prone to be treated with a total hip arthroplasty (Table 1).

### Time trends in the EQ-VAS score

**Fig. 3:**
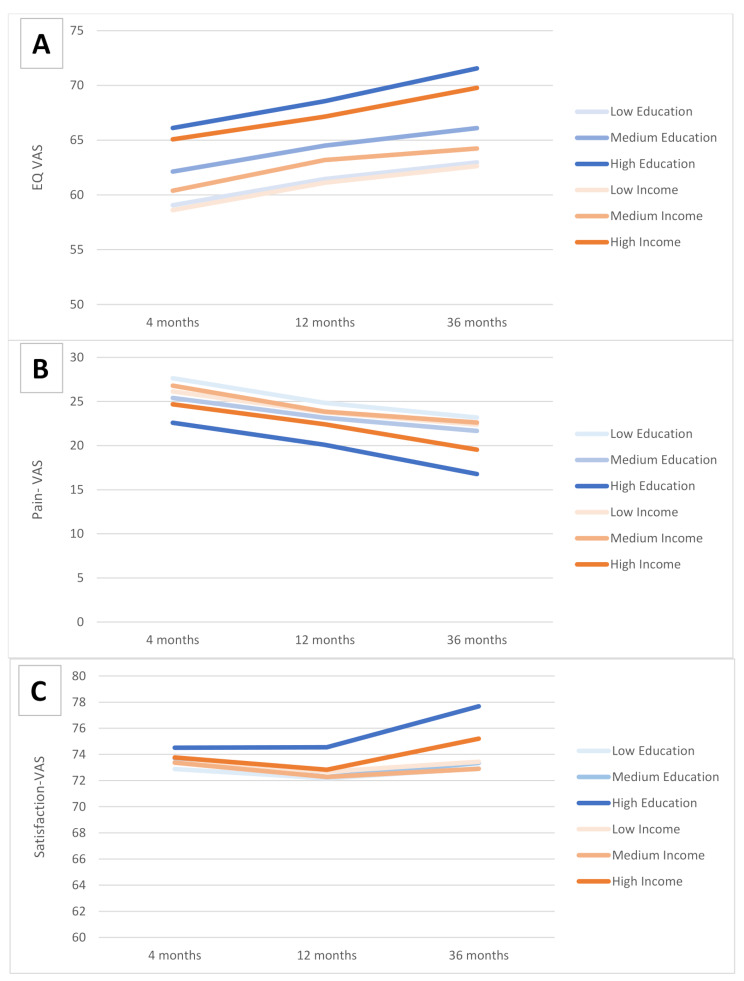
VAS scores at 4, 12, and 36 months Legend: Mean unadjusted VAS scores measured at 4, 12, and 36 months. Patients were stratified by level of education and level of household income. Panel **A** – Patient self-reported health measured by the EQ-VAS, Panel **B** – Self-reported pain - Pain-VAS, Panel **C** – Satisfaction with the operated hip – Satisfaction-VAS

The response rates for patients who were still alive and returned the questionnaire at follow-up were 57.9% at 4 months, 58.7% at 12 months, and 54.7% at 36 months. Figure 3 shows the time trends in the EQ-VAS for patients related to levels of education and income. There was a consistent improvement in the VAS score from 4 to 36 months, particularly in the high-income and high education groups. I.e. those with highest education improved by 6 points (4–36 months), while the group with the highest income improved by 5 points. There were consistently better VAS scores with higher income categories and higher education levels.

### Differences in mean VAS scores

Lower level of education and the lowest income were associated with a lower mean EQ-VAS score at 4, 12 and 36 months (Table 2). A lower level of education was associated with a greater mean Pain-VAS score at all follow-ups. The highest level of income was associated with a lower Pain-VAS score at the 12-month follow-up.

**Table 2:** Adjusted mean VAS - generalized linear model - ANCOVA

	EQ-VAS													
	4 months				12 months				36 months					
	Unadj.	Mean	95% CI	*p*	Unadj.	Mean	95% CI	*p*	Unadj.	Mean		95% CI	*p*	
Level of education														
Low	59.1	57.9	56.8 to 59.0	< 0.001	61.5	60.0	58.7 to 61.4	< 0.001	63.0	56.1		52.5 to 61.3	< 0,001	
Medium	62.1	58.7	57.7 to 59.8	0.006	64.5	61.1	59.8 to 62.5	0.001	66.1	57.8		53.5 to 62.2	< 0.001	
High	66.1	60.2	59.0 to 61.5	ref	68.6	63.1	61.6 to 64.6	ref	71.6	60.5		56.1 to 65.0	ref	
Personal income														
Low	58.6	58.0	56.8 to 59.2	0.001	61.2	60.2	59.2 to 62.1	0.023	62.6	56.8		52.4 to 61.2	< 0.001	
Medium	60.4	59.1	58.0 to 60.2	0.328	63.2	61.6	60.2 to 63.0	0.640	64.2	57.7		53.4 to 62.1	0.013	
High	65.1	59.7	58.6 to 60.8	ref	67.2	61.9	61.0 to 62.8	ref	69.8	61.6		60.2 to 62.9	ref	

	Pain-VAS													
	4 months				12 months				36 months					
	Unadj.	Mean	95% CI	*p*	Unadj.	Mean	95% CI	*p*	Unadj.	Mean		95% CI	*p*	
Level of education														
Low	27.6	26.6	25.5 to 27.6	< 0,001	24.8	25.1	23.9 to 26.4	< 0,001	23.2	23.4		19.3 to 27.5	< 0,0001	
Medium	25.4	24.6	23.6 to 25.6	< 0,001	23.2	23.4	22.2 to 24.7	< 0,001	21.7	22.1		18.1 to 26.2	< 0,0001	
High	22.6	22.0	20.8 to 23.2	ref	20.1	20.5	19.1 to 22.0	ref	16.8	17.8		13.7 to 22.0	ref	
Personal income														
Low	26.1	24.8	23.6 to 25.9	0.059	23.8	23.4	22.0 to 24.7	0.038	22.4	21.4		17.3 to 25.4	0.2539	
Medium	26.8	24.6	23.6 to 25.7	0.062	23.9	23.6	22.3 to 24.9	0.002	22.6	21.5		17.5 to 25.6	0.0791	
High	24.7	23.8	22.7 to 24.8	ref	22.4	22.2	20.9 to 23.4	ref	19.5	20.4		16.4 to 24.5	ref	

	Satisfaction-VAS													
	4 months				12 months				36 months					
	Unadj.	Mean	95% CI	*p*	Unadj.	Mean	95% CI	*p*	Unadj.	Mean		95% CI	*p*	
Level of education														
Low	72.9	73.6	72.5 to 74.6	0.211	72.2	73.0	71.4 to 74.6	0.0016	72.9	71.8		66.5 to 77.1	< 0,0001	
Medium	73.7	74.0	72.9 to 75.0	0.607	72.3	73.1	71.6 to 74.7	0.0015	73.4	72.1		66.8 to 77.4	< 0,0001	
High	74.5	74.4	73.2 to 75.7	ref	74.6	75.2	73.5 to 76.9	ref	77.7	75.9		70.5 to 81.3	ref	
Personal income														
Low	73.4	73.4	72.2 to 74.5	0.0085	72.7	73.7	72.0 to 75.3	0.5228	73.4	73.2		67.8 to 78.5	0.5228	
Medium	73.4	73.8	72.7 to 74.9	0.0489	72.3	73.4	71.7 to 75.0	0.1515	72.9	72.7		67.4 to 78.1	0.1508	
High	73.8	74.8	73.7 to 75.8	ref	72.8	74.3	72.7 to 75.9	ref	75.2	73.9		68.6 to 79.2	ref	

Legend: Unadjusted mean values of the separate VAS-scores. The adjusted values are the means from the GLM ANCOVA model adjusted for age, sex, ASA, cognitive impairment and type of surgery

Higher household income were significantly associated with higher patient satisfaction at 4 months. A higher level of education was associated with a higher patient satisfaction at the 12- and 36-month follow-ups.

### Differences in mean VAS-scores in categories of education and household income

The lowest level of education and income had a significant negative difference in mean EQ-VAS at all follow-ups. When evaluating Pain-VAS we observe that low and medium level education had significant positive difference in mean score.

**Table 3:** Differences in means - Generalized linear model - ANOVA

	EQ-VAS								
	4 months			12 months			36 months		
	Mean	95% CI	Cohen’s d	Mean	95% CI	Cohen’s d	Mean	95% CI	Cohen’s d
Level of education									
Low	-2.34	-3.54 to -1.13	0.11	-3.07	-4.41 to -1.72	0.14	-3.58	-5.19 to -1.98	0.16
Medium	-1.48	-2.60 to -0.36	0.07	-1.94	-3.20 to -0.67	0.09	-2.71	-4.19 to -1.22	0.12
High	ref			ref			ref		
Personal income									
Low	-1.72	-2.83 to -0.62	0.08	-1.38	-2.61 to -0.15	0.07	-2.51	-4.04 -0.99	0.12
Medium	-0.58	-1.53 to 0.37	0.03	-0.41	-1.48 to 0.66	0.02	-1.54	-2.82 to -0.26	0.08
High	ref			ref			ref		

	Pain-VAS								
	4 months			12 months			36 months		
	Mean	95% CI	Cohen’s d	Mean	95% CI	Cohen’s d	Mean	95% CI	Cohen’s d
Level of education									
Low	4.58	3.43 to 5.73	0.23	4.60	3.34 to 5.86	0.23	5.58	4.08 to 7.08	0.27
Medium	2.60	1.53 to 3.68	0.13	2.90	1.72 to 4.09	0.14	4.30	2.91 to 5.69	0.21
High	ref			ref			ref		
Personal income									
Low	1.02	-0.03 to 2.07	0.05	1.20	0.05 to 2.35	0.06	0.96	-0.46 to 2.37	0.05
Medium	0.87	-0.03 to 1.78	0.04	1.45	0.46 to 2.45	0.07	1.10	-0.10 to 2.29	0.05
High	ref			ref			ref		

	Satisfaction-VAS								
	4 months			12 months			36 months		
	Mean	95% CI	Cohen’s d	Mean	95% CI	Cohen’s d	Mean	95% CI	Cohen’s d
Level of education									
Low	-0.85	-2.04 to 0.33	0.04	-2.21	-3.71 to -0.71	0.10	-4.06	-5.86 to -2.26	0.18
Medium	-0.45	-1.57 to 0.66	0.02	-2.07	-3.47 to -0.67	0.09	-3.80	-5.45 to -2.15	0.17
High	ref			ref			ref		
Personal income									
Low	-1.38	-2.47 to -0.29	0.07	-0.66	-2.07 to 0.76	0.03	-0.71	-2.46 to 1.05	0.03
Medium	-0.94	-1.88 to -0.004	0.04	-0.95	-2.14 to 0.25	0.04	-1.15	-2.59 to 0.30	0.05
High	ref			ref			ref		

Legend: Mean differences in the separate VAS-scores. The GLM ANCOVA model was adjusted for age, sex, ASA, cognitive impairment and type of surgery

Patients with low and medium level of education had a negative difference in means compared to highest level at 12 and 36 months. Personal income led to significant negative difference for low and medium education at 4 months, but no significant difference at 12 and 36 months.

## Discussion

A higher level of education and higher household income were both independently associated with significantly greater self-perceived health and with less pain from the operated hip. Higher education was also associated with a significantly higher satisfaction with the results of the operation. Therefore, higher socioeconomic position was associated with better patient-reported outcome measures after hip fractures.

The mean EQ-VAS score at 4 months found in the present study is comparable to that reported in studies by Gold et al. [21], Svedbom et al. [22] and Moerman et al. [23]. Gold et al. showed that all fractures in osteoporotic patients generally reduce HRQoL measured by the EQ-VAS, and hip and spinal fractures in particular have a pronounced negative impact on HRQoL [21]. Xenodemetropoulos [24] showed that other fragility fractures have a significant negative impact on health-related quality of life (HRQoL).

Although the differences found in mean VAS scores were statistically significant, the question is whether the differences are clinically relevant. When considering an effect size measure as Cohen´s d we found that effects were small, but clearly higher for level of education compared to household income. There are no defined minimal clinically important differences (MCIDs) for the EQ-VAS for hip fractures, but the differences between levels of education and income are in the order of what Langenberger et al. [25] have documented as an MCID in hip and knee arthroplasty. It is also known that there could be a significant proportion of patients in the education and household income groups who reported a clinically important better outcome even if the group difference was smaller than the MCID [26]. There is a broad selection of methods to estimate MCID. They are not directly comparable and difficult to define in between-group studies [27]. MCID might not be appropriate to determine the relevant between-group differences measured in our study.

The impact of education and income on health-related quality of life (HRQoL) in hip fracture patients is, however, a complex issue. An important determinant of HRQL and potentially the success of surgery in this age group is the presence of preexisting health conditions. In this study we included ASA class and presence of cognitive impairment as measures of preexisting comorbidity. We observed higher comorbidity in groups of lower education and income, and adjusted for these factors in the analyses. Suriyawongpaisal et al. [28] reported that while comorbidities negatively affected HRQoL, age, sex, income, and education level did not. Greene et al. [29] reported that higher education was associated with greater HRQoL and less pain after total hip artrhoplasty. Similarly, Valentin et al. reported that low education was associated with reduced post fracture HRQoL, especially for the mental component score [30]. Griffin and coworkers [31] demonstrated that national standards of best practices were associated with improved HRQoL outcomes, and that patients with lower SEP may receive best practice care less often compared to patients with higher SEP.

Education and income may not directly impact HRQoL in hip fracture patients, but they can influence access to rehabilitative care that improves HRQoL. This “health literacy” [32] refers to an individual’s ability to obtain, process, and understand basic health information and services needed to make appropriate and well-informed health decisions. Surgeons and other healthcare providers might also insufficiently tailor their information provision to patients with lower health literacy. Higher education levels are often associated with higher health literacy, as education provides individuals with the skills and knowledge to improve their ability to understand health information, adhere to treatment plans, and engage in healthy behaviors [32, 33]. In a review, Petrovic et al. [34] showed that health behaviors explained a large proportion of the SEP-health gradient in studies conducted in North America and Northern Europe. This is supported by Olsen et al. [35], who also concluded that variations in health and well-being are explained by healthy behaviors rather than by educational attainment. Spronk et al. [36] showed that education-dependent inequities in HRQoL in three national populations were explained by the presence of chronic health conditions and inability to work. Thus, education level and income might be proxies for health behavior and general health differences.

The mechanism for SEP to impact HRQoL is likely multifactorial, and the available variables cannot fully explain this. We have shown lower comorbidity among individuals with higher education or income which can lead to better access to healthcare services, medications, and support, leading to better outcomes. In addition, it can be assumed that higher SEP also affects access to physiotherapy and other rehabilitation services which again might affect HRQoL positively [37]. Additionally, differences in reporting rates and reporting practices may play a role [38].

### Strengths and limitations

We included 35,206 out of 39,690 patients (89%) in a national hip fracture population in this observational cohort study. There are very few high-quality studies that include more than 1,500 individuals with hip fractures, and most research reporting PROM data have had small patient populations [39]. Few studies have provided follow-up data longer than one year. There are no comparable population data, as no other hip fracture registries have regularly obtained PROMs from hip fracture patients. Selection, attrition, and a low response rate, together with the effect of the covariates on the outcome measures, might lead to an overestimation of HRQoL in observational data and is important to concider when interpreting PROMs from this patient group. In a previous study on this cohort [38] we demonstrated these effects and knowledge from this project was important to integrate in the present study. Exclusion of 2009 patients with bilateral fractures in the study period introduces some selection bias, but was considered to affect results less than the effect of a second fracture during follow-up for the first fracture. We contend that, after accounting for the biases and adjusting for the factors most susceptible to lead to overestimation, the data shown here produces accurate and reproducible results.

The significant differences in mean VAS scores between SEP-groups demonstrated in this study might be a result of a group difference prefracture (i.e., baseline), but such data were not available in this study. In the adjusted analyses, differences were smaller. Thus, the results must be interpreted with this in mind. The Pain-VAS and Satisfaction-VAS are not validated; thus, there are no defined MCIDs for these outcome measures, but they follow the same temporal and group patterns as the validated EQ-VAS score.

This was an observational cohort study, and we cannot make inferences of causality. DAGs allow researchers to identify and distinguish different causal pathways through which variables may influence each other and ensure that we predict the best possible causal inference, fully aware that there still might be residual confounding.

## Conclusions

In summary, we found that a higher level of education and higher household income were both independently and significantly associated with less pain from the operated hip and greater self-perceived health. Higher education was also significantly associated with better satisfaction with the result of the operation. Therefore, socioeconomic factors may affect PROMs after hip fractures.

However, it is important to note that the association between socioeconomic factors and PROMs may be partly mediated by differing health behaviors and levels of health literacy. Disparities in SEP and the promotion of health literacy should be considered integral components of interventions aimed at improving PROMs following hip fracture surgery.

## Data Availability

No datasets were generated or analysed during the current study.

## Abbreviations

ASA: American Society of Anesthesiologists physical status class
DAG: Directed Acyclic Graph
HRQoL: Health Related Quality of Life
NHFR: Norwegian Hip Fracture Register
PROM: Patient Reported Outcome Measures
SEP: Socioeconomic position
SN: Statistics Norway
VAS: Visual Analog Scale
